# Porcine IFI16 Negatively Regulates cGAS Signaling Through the Restriction of DNA Binding and Stimulation

**DOI:** 10.3389/fimmu.2020.01669

**Published:** 2020-08-14

**Authors:** Wanglong Zheng, Rongyun Zhou, Shuangjie Li, Shan He, Jia Luo, Meiqin Zhu, Nanhua Chen, Hongjun Chen, François Meurens, Jianzhong Zhu

**Affiliations:** ^1^Comparative Medicine Research Institute, Yangzhou University, Yangzhou, China; ^2^College Veterinary Medicine, Yangzhou University, Yangzhou, China; ^3^Jiangsu Co-innovation Center for Prevention and Control of Important Animal Infectious Diseases and Zoonoses, Yangzhou, China; ^4^Joint International Research Laboratory of Agriculture and Agri-Product Safety, Yangzhou, China; ^5^Shanghai Veterinary Research Institute, Chinese Academy of Agriculture Sciences, Shanghai, China; ^6^BIOEPAR, INRAE, Oniris, Nantes, France

**Keywords:** innate immunity, DNA sensor, signaling, porcine, mutual relationship

## Abstract

The innate immunity DNA sensors have drawn much attention due to their significant importance against the infections with DNA viruses and intracellular bacteria. Among the multiple DNA sensors, IFI16, and cGAS are the two major ones, subjected to extensive studies. However, these two DNA sensors in livestock animals have not been well defined. Here, we studied the porcine IFI16 and cGAS, and their mutual relationship. We found that both enable STING-dependent signaling to downstream IFN upon DNA transfection and HSV-1 infection, and cGAS plays a major role in DNA signaling. In terms of their relationship, IFI16 appeared to interfere with cGAS signaling as deduced from both transfected and knockout cells. Mechanistically, IFI16 competitively binds with agonist DNA and signaling adaptor STING and thereby influences second messenger cGAMP production and downstream gene transcription. Furthermore, the HIN2 domain of porcine IFI16 harbored most of its activity and mediated cGAS inhibition. Thus, this study provides a unique insight into the porcine DNA sensing system.

## Introduction

The innate immune system acts as the first line of host defense and senses multiple danger signals from pathogens by recognizing the pathogen-associated molecular patterns (PAMPs) (1). It also detects damage-associated molecular patterns (DAMPs) to maintain homeostasis (1, 2). The PAMPs and DAMPs are both recognized by innate immune pattern recognition receptors (PRRs), which include Toll-like receptors (TLRs), RIG-I-like receptors (RLRs), NOD-like receptors (NLRs), C-type lectin like receptors (CLRs), and cytosolic DNA receptors (CDRs). Upon activation, the PRRs trigger intracellular signaling to initiate either gene transcription or protease-dependent cytokine secretion, resulting in the production of anti-viral interferons (IFNs), proinflammatory cytokines, and chemokines to directly combat pathogens and shape subsequent adaptive immunity.

The DNA sensors consist of a broad range of receptors, including membrane-bound TLR9 and various CDRs. TLR9 was the first identified DNA sensor localized in the ER/endosome. It recognizes endolysosomal under-methylated CpG DNA to activate transcription factors IRF7 and NF-κB and stimulate type I IFN production (3). TLR9 expression is immune cell specific, mainly expressed in plasmacytoid dendritic cells (pDCs) and B cells. Two CDRs, DExD/H-box helicases DHX36 and DHX9, are required in pDCs for TLR9-dependent IFNα and TNF-α productions, respectively, thus possible accessory receptors for TLR9 (4, 5). In addition to DHX36 and DHX9, CDRs include also DAI, AIM2, RNA Pol III, LRRFIP1, IFI16, DDX41, DNA-PK, MRE11, cGAS, and STING (5).

DAI (or ZBP-1) was the first discovered CDR, able to induce IFNs through IRF3 and NF-κB activations (6). However, DAI knockout mice demonstrated normal DNA-mediated cytokine production; thus, the role of DAI as a DNA sensor has been controversial and it also indicated the existence of other CDRs (7). AIM2 belongs to PYHIN family proteins containing Pyrin and HIN domains. It binds viral double-stranded (ds) DNA using the C-terminal HIN domains to subsequently recruit downstream adaptor ASC via its N-terminal Pyrin domain through homotypic interaction. Further, ASC is able to recruit and activate caspase-1 by a homotypic CARD domain interaction to formulate inflammasome. In turn, caspase-1 causes proteolytic cleavage and maturation of the proinflammatory cytokines IL1β and IL18 (5, 8, 9). RNA polymerase III (Pol III) was described as a DNA sensor because of the transcription of AT-rich dsDNA, such as poly (dA:dT), into 5-triphosphate RNA, which can then activate RIG-I leading to IFNβ induction (10, 11). LRRFIP1 was reported to bind both dsDNA and dsRNA, and then interact with and activate β-catenin, which increases IFNβ expression as a co-activator by binding with IRF3 and recruiting the acetyltransferase p300 to the IFNβ enhanceosome (12). DDX41, an additional DExD/H-box helicase, was shown to bind with DNA and activate STING/TBK1-dependent IRF3 and NF-κB, and subsequent cytokine production (13). Besides dsDNA from transfection and virus infection, DDX41 was further reported to bind with bacterial cyclic dinucleotides (CDNs), cyclic-di-GMP, and cyclic di-AMP to activate similar downstream signaling (14). DNA-PK and MRE11 are both nuclear DNA damage sensor proteins, with the former comprising heterocomplex of Ku70, Ku80, and the catalytic subunit DNA-PKcs. Both protein complexes in cytosol were reported to be involved in DNA sensing and trigger STING-dependent cytokine production (15, 16).

STING (also called MITA, MPYS, and ERIS) was discovered in 2008 by several groups independently (17–20). STING acts as the signaling adaptor for DNA sensing pathways even though there are reports of its direct DNA sensing (21). Furthermore, STING also directly recognizes CDNs such as bacterial c-di-GMP and mammalian second messenger cGAMP to induce a type I IFN response (22, 23). IFI16 was the first reported STING-dependent DNA sensor in 2010 and is also a PYHIN family protein mediating IFN induction (24). IFI16 at steady state is present in the cell nucleus, but also shuttles between nucleus and cytosol depending on the acetylation status of its nuclear localization sequence (25). Moreover, nuclear IFI16 also engages in inflammasome formation (26), but the mechanisms by which IFI16 initiates signal to both STING and inflammasome are still unknown (5). cGAS was identified in 2013 as a cytosolic DNA sensor; upon DNA stimulation, it utilized substrates ATP and GTP to synthesize second messenger 2′5′-cGAMP, which directly activates STING signaling (27). Since its discovery, the cGAS-cGAMP-STING pathway has been subjected to extensive studies (28).

IFI16 and cGAS are the most extensively studied and best-characterized DNA sensors. However, these two DNA sensors are not well defined in livestock animals. In this study, we investigated porcine DNA sensors IFI16 and cGAS. Our study found that both porcine DNA sensors trigger STING-dependent signaling, and IFI16 negatively regulates cGAS signaling to IFN mainly through competitive binding to agonist DNA and adaptor STING.

## Materials and Methods

### Cells and Reagents

HEK-293T and porcine kidney-15 (PK15) cells were cultured in Dulbecco's modified Eagle medium (DMEM, Hyclone Laboratories, USA) containing 10% fetal bovine serum (FBS) and 100 IU/ml of penicillin plus 100 μg/ml streptomycin. Porcine alveolar macrophages (PAMs) were cultured in Roswell Park Memorial Institute 1640 (RPMI, Hyclone) containing 10% FBS with penicillin/streptomycin. All cells were maintained at 37°C with 5% CO_2_ in a humidified incubator. Restriction endonucleases, Phusion Hot Start High Fidelity DNA polymerase (M0203S), and T4 DNA ligase (M0203S) were all purchased from New England Biolabs (Beijing, China). Gateway™ LR Clonase™ II Enzyme mix, Lipofectamine™ 2000, and Goat Anti-Mouse IgG (H+L) Antibody DyLight 488 were from ThermoFisher Scientific (Shanghai, China). Pro Ligation-Free Cloning Kit (Cat No: E086/E087) was from Applied Biological Materials Inc. (Richmond, Canada). TRIpure Reagent for RNA extraction was from Aidlab (Beijing, China). EasyScript Reverse Transcriptase, 2 × EasyTag PCR SuperMix, BluePlus Protein Marker, anti-HA mAb, anti-FLAG mAb, anti-GFP mAb, anti-Actin mAb, HRP anti-mouse IgG, HRP anti-rabbit IgG, and TransDetect Double-Luciferase Reporter Assay Kit were bought from Transgen Biotech (Beijing, China). The anti-HA rabbit pAb and anti-FLAG rabbit pAb were from Sangon Biotech (Shanghai, China). The anti-p-TBK1 (D52C2) and anti-p-IRF3 (4D4G) were from Cell Signaling Technology (Danvers, MA, US). Phanta Max Super-Fidelity DNA Polymerase, PCR Purification Kit, Gel Extraction Kit, Plasmid Mini-prep Kit were from Vazyme Biotech Co., Ltd. (Nanjing, China). Forty-five-base pair (45-bp) dsDNA (TACAGATCTACTAGTGATCTATGACTGATCTGTACATGATCTACA) as a DNA agonist was synthesized by GENEWIZ (Shouzhou, China). HSV-1 (KOS strain, whose VP26 was fused with GFP) was a gift from Dr. Tony Wang in SRI International USA. The second messenger or STING agonist 2′3′-cGAMP was bought from InvivoGen (Hong Kong, China).

### Molecular Cloning and Gene Mutations

Total RNA was extracted from primary PAMs using TRIzol^®^ reagent (ThermoFisher Scientific). From the total RNA, porcine cGAS (XM_013985148) and IFI16 (XM_013996900) open reading frames (ORFs) were amplified by RT-PCR using the designed primers shown in Supplementary Table 1. The PCR products were digested with *Nco*I/*EcoR*V and *Sal*I/*EcoR*V, respectively, and cloned into the corresponding sites of the Gateway entry vector pENTR4-2HA, which was adapted from pENTR4 (Addgene) by inserting a 2HA sequence behind *EcoR*V to express C-terminal HA tagged genes. The sequence confirmed that HA-tagged pcGAS and pIFI16 were transferred from pENTR4 vectors to Destination vectors pDEST47 (Addgene) by LR recombination to obtain the final pcDNA recombinant expression vectors. If not specifically mentioned, cGAS and IFI16 in this paper refer to porcine cGAS and porcine IFI16, respectively.

For IFI16 subcloning and mutations, the Pyrin1, HIN1, Pyrin2, HIN2, ΔPyrin1, and ΔHIN2 fragments were amplified by PCR using Phanta Max Super-Fidelity DNA Polymerase from IFI16 plasmid template using the designed primer pairs shown in Supplementary Table 1. For IFI16 deletion mutants ΔHIN1 and ΔPyrin 2, the two fragments flanking the deletion site were amplified by PCR from IFI16 plasmid; next, the two flanking fragments together with the Bridge fragment were joined together by the fusion PCR. The Pyrin1, HIN1, Pyrin2, and HIN2 domain fragments were digested with *Nhe*I and *EcoR*V and cloned into pcDNA3.1 vector expressing C-terminal 2HA as we described previously (29), whereas the deletion fragments ΔPyrin1, ΔHIN1, ΔPyrin 2, and ΔHIN2 were cloned into the same sites of the above pcDNA3.1-2HA vector using the Pro Ligation-Free Cloning Kit.

### CRISPR gRNA Design, gRNA Expressing Lentiviruses, and Stable KO Cells

The CRISPR gRNAs targeting porcine cGAS and IFI16 were designed using the web tool from Benchling (www.benchling.com). For porcine cGAS and IFI16, three gRNAs were chosen based on the predicted high scores, respectively, and the encoding DNA sequences are shown in Supplementary Table 2. The annealed gRNA encoding DNA pairs were ligated with *BsmB1*-digested lentiCRISPRv2 vector (Addgene), and the efficacy and specificity of these gRNA expressing lentiviral vectors targeting porcine cGAS and IFI16 were demonstrated as shown in Supplementary Figures 2, 3. The gRNA expressing lentiviruses were generated by co-transfecting lentiCRISPRv2-gRNAs with package plasmids psPAX2 and pMD2.G into 293T cells using Lipofectamine 2000. The supernatants containing three gRNA expressing lentiviruses were mixed equally and used to infect the PK15 cells and PAMs, respectively. Then, the infected PK15 cells were selected with 1.5 μg/ml puromycin, whereas infected PAMs were selected with 1 μg/ml puromycin. After puromycin selection, the CRISPR vector control, pcGAS KO, IFI16 KO stable PAMs, and CRISPR vector control, cGAS KO, and IFI16 KO stable PK15 cells were all prepared and ready for use.

### DsDNA Binding Assay and Co-immunoprecipitation

The 5′-biotin-labeled 45-bp dsDNA was also synthesized and obtained from GENEWIZ (Shouzhou, China). Streptavidin Agarose (Cat No: S951, ThermoFisher Scientific) was washed three to five times with PBS by centrifugation at 10,000 *g* and suspended in PBS. Each milliliter of streptavidin agarose was mixed with 20 nmol 5′-biotin-labeled 45-bp dsDNA, incubated at RT for 30 min, and washed three to five times with PBS, and the resultant 45-bp dsDNA-agarose was stored at 4°C for protein pull-down assay. For protein pull-down assay, cells in a six-well plate (8 × 10^5^ cells/well) were transfected for 48 h, harvested, and lysed in 500μl of RIPA buffer (50 mM Tris, pH 7.2, 150 mM NaCl, 1% sodium deoxycholate, and 1% Triton X-100) containing protease inhibitors on ice for 30 min. The 50 μl of cleared lysate was used as input control, and the remainder was incubated with 20 μl of dsDNA-agarose at 4°C overnight with shaking. Next day, the dsDNA agarose was washed three times by centrifugation, and bound proteins were eluted with 20 μl of 2 × SDS sample buffer by heating at 100°C for 10 min. The elution supernatants from centrifugation together with input controls were subjected to Western blot analysis. For co-immunoprecipitation, the cleared cell lysate from transfected cells was incubated with 1 μg of specific antibody at 4°C overnight with shaking and further incubated with Protein A/G PLUS-Agarose (sc-2003, Santa Cruz Biotechnology) for 2–3 h. The agarose was similarly washed and eluted with 20 μl of 2 × SDS sample buffer. The elution and input were both subjected to Western blot analysis.

### Western Blot Analysis

Cell lysates or precipitated samples were resolved on 10% SDS-polyacrylamide gels in the presence of 2-mercaptoethanol. The protein bands on gels were transferred onto PVDF membranes and the membranes were blocked with 5% non-fat dry milk Tris-buffered saline, pH 7.4, with 0.1% Tween-20 (TBST), incubated with various primary antibodies. After washing with TBST, the membranes were incubated with HRP-conjugated goat anti-mouse IgG or goat anti-rabbit IgG (1:10,000 dilutions). The bound secondary antibody signals were detected with enhanced chemiluminescence (ECL) substrate (Tanon, China) and visualized by Western blot imaging system (Tanon, China).

### Fluorescence Microscopy

PAMs grown on coverslips in 24-well culture plate (1 × 10^5^ cells/well) or 293T cells grown on coverslips in 12-well plates (4 × 10^5^ cells) were transfected with pcGAS-HA, pIFI16-HA, and GFP-pSTING plasmids, respectively, using Lipofectamine 2000. Forty-eight hours later, the transfected pcGAS and pIFI16 cells on coverslips were fixed with 4% paraformaldehyde at RT for 15 min and permeabilized with 0.5% Triton X for 10 min. After washing with PBS, the cells were sequentially incubated with primary anti-HA mAb (1:500) and goat anti-mouse IgG (H+L) DyLight 488 second antibody (1:200). The stained cells and fixed GFP-pSTING cells were counterstained with 0.5 μg/ml 4′,6-diamidino-2-phenylindole (DAPI, Beyotime, China) at 37°C for 15 min to stain the cell nucleus and the coverslips sealed with nail polish. Lastly, the 293T cells were visualized under fluorescence microscope (Olympus, Japan) and the PAMs were observed under laser-scanning confocal microscope (LSCM, Leica SP8, Solms, Germany) at the excitation wavelengths 340 and 488 nm, respectively.

### Promoter-Driven Luciferase Reporter Gene Assays

293T cells grown in 96-well plates (3 × 10^4^ cells/well) were co-transfected by Lipofectamine 2000 with ISRE-luciferase reporter or ELAM (NF-κB)-firefly luciferase (Fluc) reporter (10 ng/well) and β-actin *Renilla* luciferase (Rluc) reporter (0.2 ng/well), together with the indicated plasmids or vector control (5–40 ng/well). The total DNA per well was normalized to 50 ng by adding empty vector. About 36 h post-transfection, the cells were harvested and lysed, and both Fluc and Rluc activities were sequentially measured using the TransDetect Double-Luciferase Reporter Assay Kit. The results were expressed as fold induction of ISRE or ELAM (NF-κB)-Fluc compared with that of vector control after Fluc normalization by corresponding Rluc.

### RT-PCR and Quantitative RT-PCR

293T, PAMs, or PK15 cells grown in 24-well plates (3 × 10^5^ cells) were subjected to different treatments. The treated cells were harvested and RNA extracted with TRIpure Reagent. The extracted RNA was reverse transcribed into cDNA with EasyScript Reverse Transcriptase, and then the target gene expressions were measured by PCR or quantitative PCR with 2 × EasyTag PCR SuperMix by using StepOnePlus equipment (Applied Biosystems). The PCR program is denaturation at 94°C for 30 s followed by 25 cycles of 94°C for 5 s, 60°C for 30 s, and 72°C for 30 s, whereas the qPCR program is denaturation at 94°C for 30 s followed by 40 cycles of 94°C for 5 s and 60°C for 30 s. The PCR primers for hIFN-β, hISG56, hIL8, hRPL32, pIFNβ, pISG56, pISG60, pIL-8, and pβ-actin are shown in Supplementary Table 3. The PCR products were analyzed by agarose gel electrophoresis and visualized by imaging, whereas in qPCR, the transcriptional levels of IFN-β, ISG56, ISG60, and IL-8 were calculated using ΔΔC_T_ method.

### Statistical Analysis

All the experiments are representative of three similar experiments and the representative experimental data in graphs were shown as the mean ± SD of duplicate wells. The statistical analysis was performed with the Student *t*-test or one-way ANOVA where appropriate, which are built within the software GraphPad Prism 5.0.

## Results

### Characterization of Porcine cGAS and IFI16 Signaling Activity

**Figure 1:**
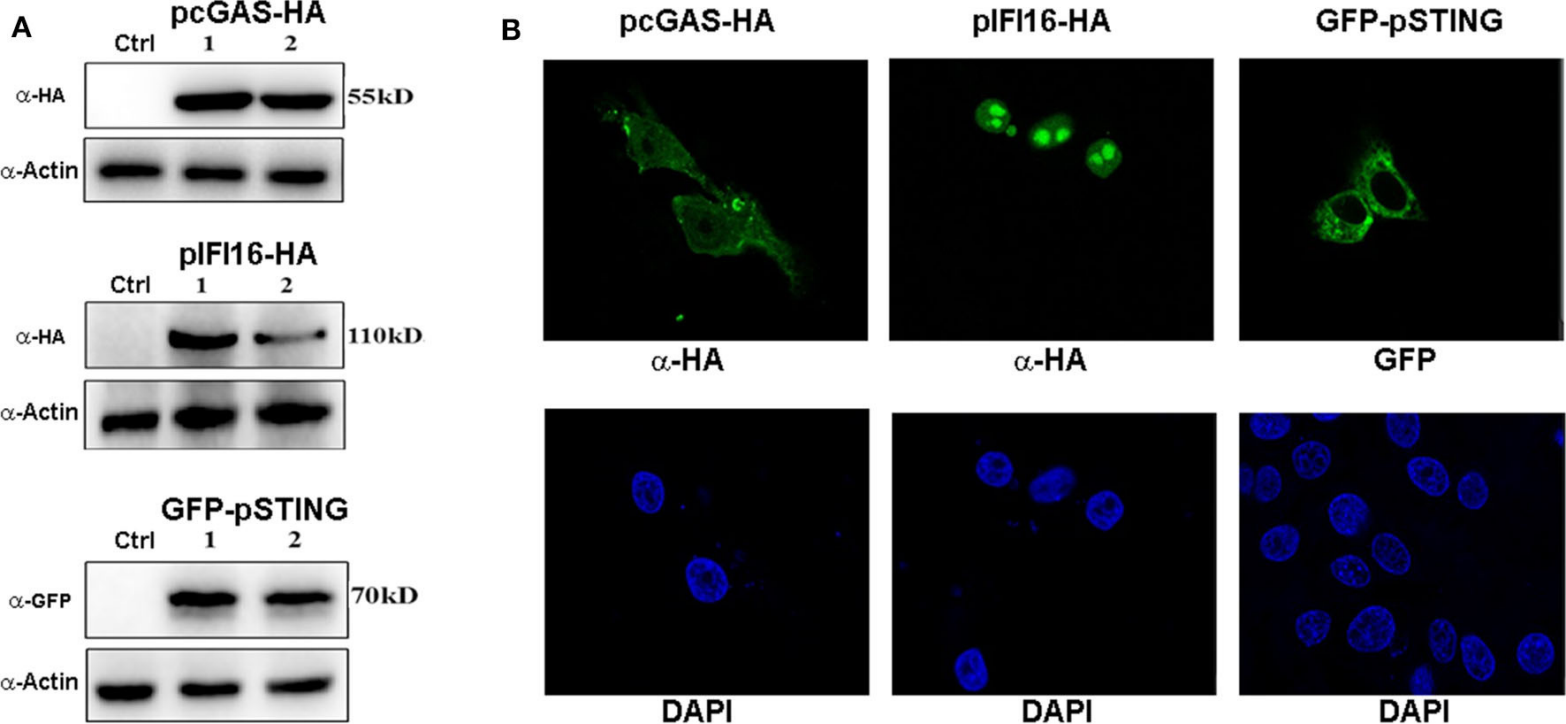
The expressions of porcine DNA sensors cGAS, IFI16, and STING. **(A)** The pcDNA-cGAS, pcDNA-IFI16, pEGFP-STING, and control plasmids (0.5 μg each) were transfected into 293T cells (4 × 10^5^ cells/well in a 24-well plate) using the Lipofectamine 2000. Forty-eight hours post-transfection, the protein expressions were detected by Western blotting with the indicated antibodies. Numbers 1 and 2 denote the two expression clones. **(B)** PAMs on coverslips in a 24-well plate (1 × 10^5^ cells/well) were transfected with pcDNA-cGAS, pcDNA-IFI16, and pEGFP-STING as in **(A)**, and the fixed and stained PAMs were examined by confocal microscopy.

The porcine cGAS has an amino acid (AA) sequence identity of 75.13% to human cGAS, while porcine IFI16 has only 45.61% identity of AA to human IFI16. Considering the low sequence identities of the two receptors between porcine and human, it is important to investigate the signaling functions of the two porcine receptors especially porcine IFI16. To study the signaling functions of the two porcine DNA receptors, the porcine cGAS and IFI16 gene cDNAs were amplified and both cloned into the Gateway pENTR4-2HA vector. Following sequence confirmation, cDNAs were further transferred into destination pcDNA expression vectors by LR reaction. The pcDNA expression constructs of porcine cGAS and IFI16 were transfected into 293T cells and PAMs, and their expressions were examined by Western blotting and IFA using the anti-HA antibody. As shown in Figure 1 and Supplementary Figure 1, the porcine cGAS was expressed as a 55-kD protein, mainly localized in the cytoplasm similar to previously reported (27, 30). Conversely, the porcine IFI16 was expressed predominantly in cell nucleus, as a 110-kD protein. We also examined the expression of signaling adaptor porcine STING we previously cloned in pEGFP-C1 vector, and the GFP-STING fusion protein with M.W. of 70 kD was localized in the cytoplasm.

**Figure 2:**
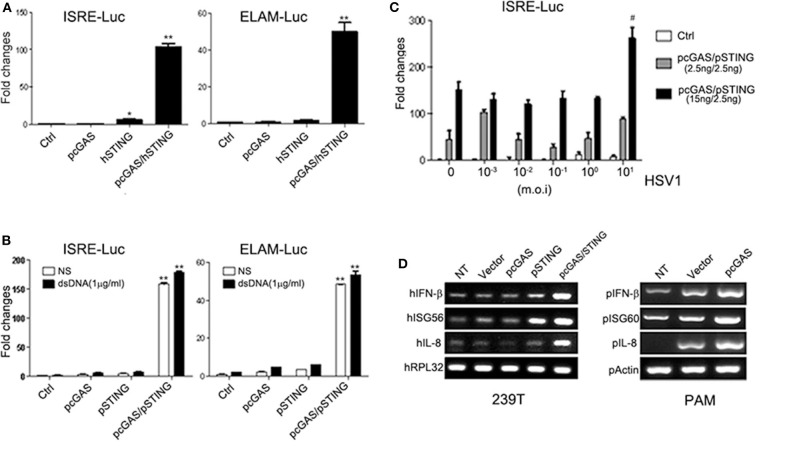
Characterization of porcine DNA sensor cGAS. **(A)** 293T cells grown in 96-well plates were transfected with pcGAS (20 ng), hSTING (10 ng) plus ISRE-Fluc/ELAM-Fluc (10 ng), and Rluc (0.2 ng), which was normalized to 50 ng/well. Twenty-four hours post-transfection, the luciferase activities were measured with Double-Luciferase Reporter Assay. **(B)** 293T cells were transfected as in **(A)** with pSTING replacing hSTING, and 24 h later were mock stimulated (NS) or stimulated with dsDNA by transfection. Twelve hours post-stimulation, the luciferase activities were measured. **(C)** 293T cells were transfected as indicated, and 24 h later stimulated with HSV-1. Twelve hours post-stimulation, luciferase activities were measured. **(D)** 293T cells and PAM grown in 24-well plates were not transfected (NT) or transfected with 0.5 μg each recombinant plasmid and 24 h later the downstream gene transcription was examined by RT-PCR. ^*^p < 0.05, ^**^p < 0.01 vs. controls. #*p* < 0.05 vs. non-HSV-1 stimulation.

The signaling function of porcine cGAS was first examined together with the functionally known human adaptor STING. Porcine cGAS alone did not show any activity in either ISRE promoter or ELAM (NF-κB) promoter assay whereas human STING alone had weak ISRE activity. When porcine cGAS was transfected together with human STING, it showed strong activity in both ISRE and NF-κB promoter assays (Figure 2A). Next, the porcine cGAS was tested with porcine STING, which gave similar results (Figure 2B). Upon dsDNA agonist treatment, the porcine cGAS/STING activity had a modest increase in both ISRE and NF-κB promoter assays (Figure 2B). When stimulated with HSV-1, the porcine cGAS/STING activity showed significant increase in ISRE promoter assay at the high concentration of virus (Figure 2C). The downstream gene inductions including IFNβ, ISG56, and IL8 were obvious in porcine cGAS/STING transfected 293T cells, which normally lack both protein expressions (left panel, Figure 2D). In PAMs, the transfection of porcine cGAS alone was sufficient to induce downstream IFNβ, ISG60, and IL8 productions (right panel, Figure 2D).

**Figure 3:**
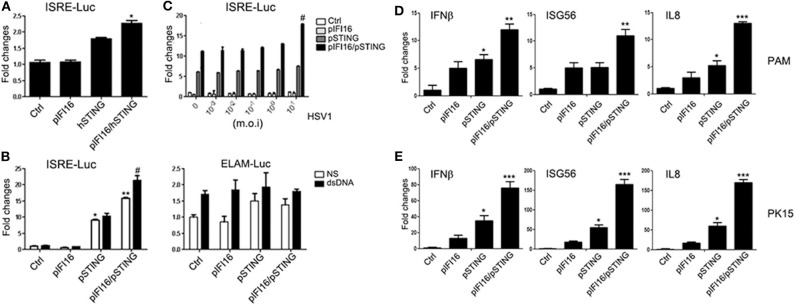
Characterization of porcine DNA sensor IFI16. **(A)** 293T cells grown in 96-well plates were transfected with pIFI16 (20 ng), hSTING (10 ng) plus ISRE-Fluc (10 ng), and Rluc (0.2 ng), which was normalized to 50 ng/well. Twenty-four hours post-transfection, the luciferase activities were measured. **(B)** 293T cells were transfected as in **(A)** using ISRE-Fluc (*left*) or ELAM-Fluc (*right*) with pSTING replacing hSTING, and 24 h later, the cells were stimulated with 1 μg/ml dsDNA by transfection. Twelve hours post-stimulation, the luciferase activities were measured. **(C)** 293T cells were transfected as in **(B)**, and 24 h later stimulated with HSV-1. Twelve hours post-stimulation, luciferase activities were measured. **(D,E)** PAMs and PK15 cells grown in 24-well plates were transfected with 0.5 μg of each recombinant plasmid, and 24 h later, the downstream gene transcription was examined by RT-qPCR. ^*^p < 0.05, ^**^p < 0.01, ^***^p < 0.001 vs. controls. #*p* < 0.05 vs. non-HSV-1 stimulation.

Porcine IFI16 alone did not have any activity in promoter assay in 293T cells. When co-transfected with either human STING or porcine STING, the porcine IFI16/STING exhibited ISRE promoter activity but not as strong as that of porcine cGAS/STING (Figures 3A,B). The porcine IFI16/STING ISRE activity was significantly upregulated by dsDNA (left panel, Figure 3B) and by high titers of HSV-1 (Figure 3C). In the NF-κB promoter assay, porcine IFI16/STING showed no activity due to its weak signaling activity (right panel, Figure 3B). The downstream gene inductions of IFNβ, ISG56, and IL8 were observed in both PAMs and PK15 cells transfected with porcine IFI16 alone, porcine STING alone, or both (Figures 3D,E).

### The Porcine IFI16 Interferes With cGAS Signaling and Downstream Gene Transcription

**Figure 4:**
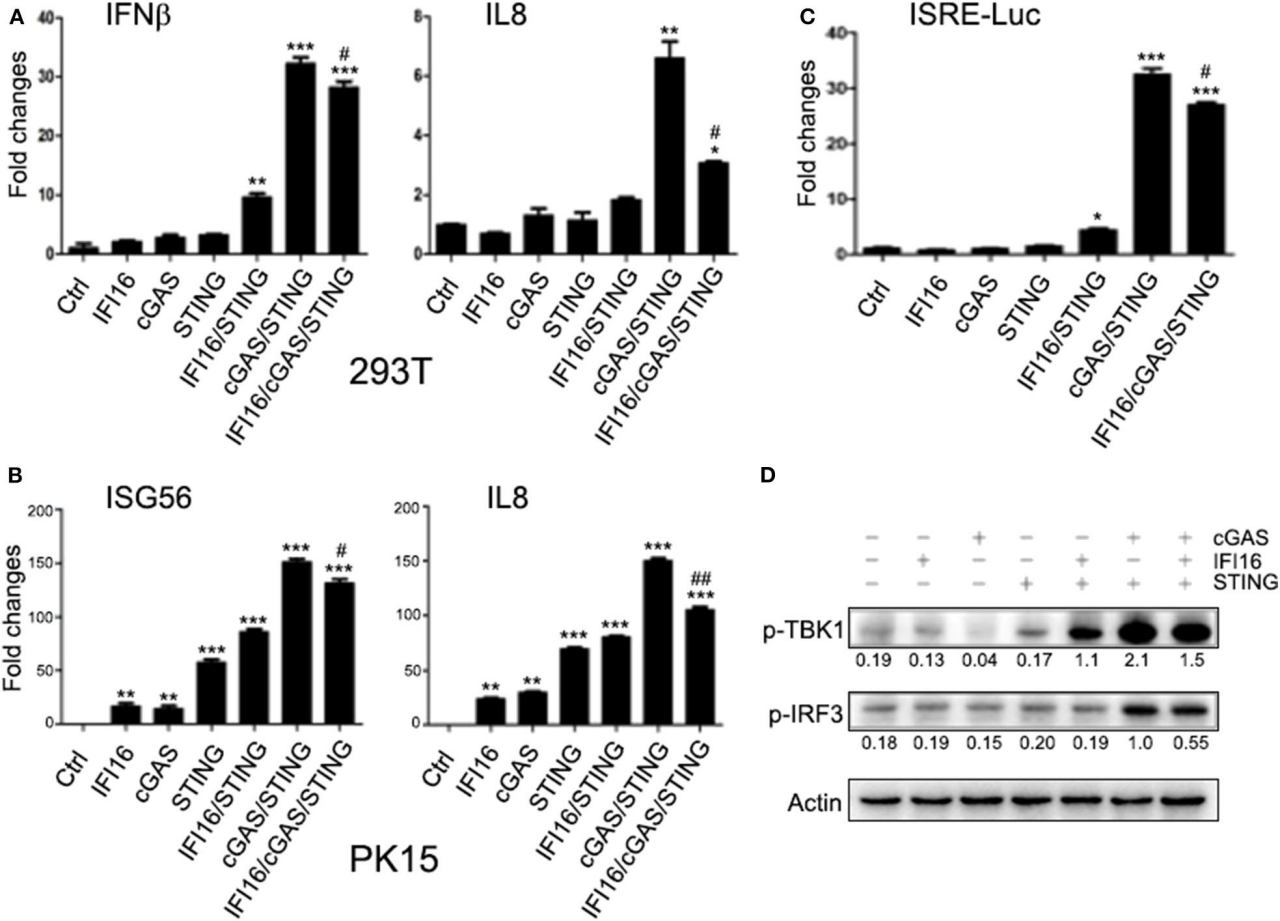
Effects of ectopic porcine IFI16 on porcine cGAS-STING signaling. 293T **(A)** and PK15 cells **(B)** in a 24-well plate were transfected with 0.5 μg of each plasmid, and normalized to 1.5 μg of total DNA each well. Next day, cells were subjected to RT-qPCR analysis. **(C)** 293T cells in a 96-well plate were transfected with the indicated combinations of porcine cGAS (5 ng), IFI16 (20 ng), and STING (2 ng) plus Fluc and Rluc reporters. Twenty-four hours post-transfection, the cells were examined for Fluc and Rluc activities. **(D)** 293T cells (5 × 10^5^ cells/well in a 24-well plate) were transfected with 0.5 μg of each plasmid, and normalized to 1.5 μg each well. Forty-eight hours later, the cells were subjected for Western blot analysis. The densitometry values of p-TBK1 and p-IRF3 after normalization by actin were indicated at the bottom of the bands. ^*^p < 0.05, ^**^p < 0.01, ^***^p < 0.001 vs. controls. #*p* < 0.05, ##*p* < 0.01 vs. cGAS/STING samples.

Several previous studies on human and mouse cGAS and IFI16 investigated their mutual relationship and revealed the cooperation between these two DNA receptors (30–34). To understand whether the two porcine DNA receptors also have cooperative relationship, we first co-transfected IFI16 with cGAS/STING in 293T cells and PK15 cells and examined the downstream gene transcription by RT-qPCR. As shown in Figures 4A,B, the downstream expressions of IFNβ and IL8 genes in 293T and ISG56 and IL8 genes in PK15 (activated by cGAS/STING) were both significantly decreased with IFI16 when compared with those without IFI16. The results indicated that porcine IFI16 does not promote cGAS signaling; instead, it inhibits cGAS activity. Next, we observed a similar inhibition of cGAS/STING ISRE activity by IFI16 in the promoter assay (Figure 4C). We also monitored the activation of DNA signaling pathway molecules with or without IFI16 using Western blotting; it turned out that the phosphorylated TBK1 (p-TBK1) and IRF3 (p-IRF3) activated by cGAS/STING were both slightly downregulated by IFI16 (Figure 4D).

**Figure 5:**
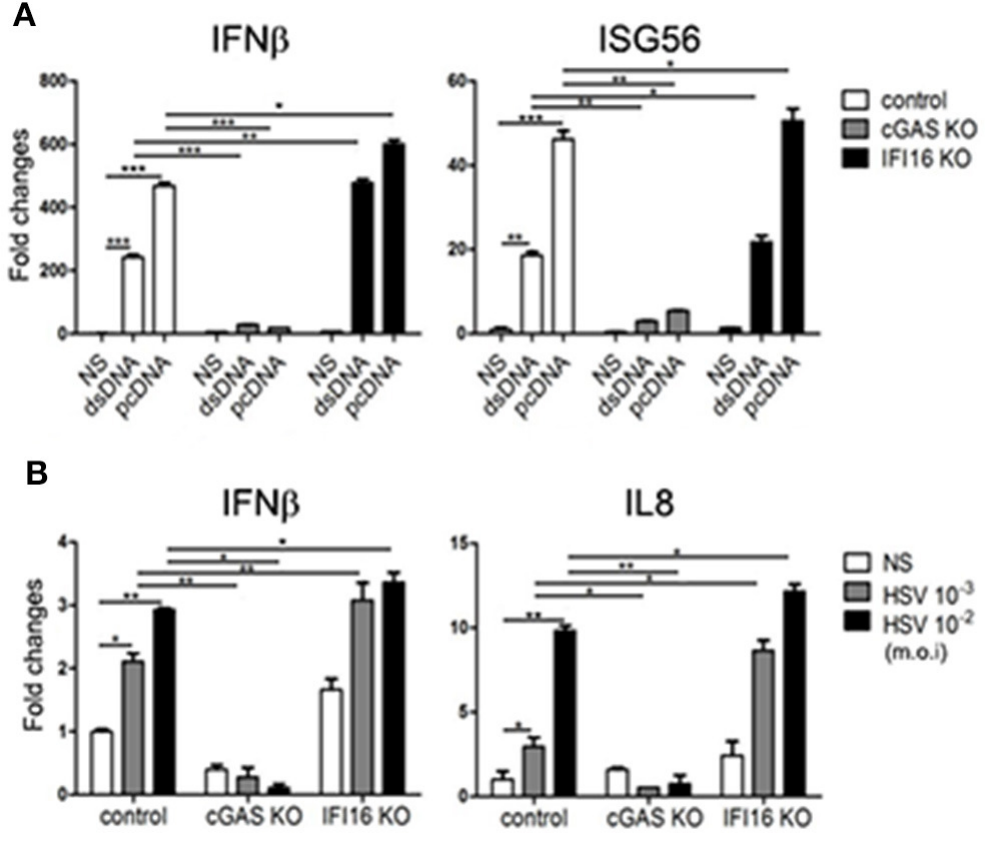
Effects of IFI16 and cGAS KO on DNA and HSV-1 stimulated gene transcription. PAM cGAS KO, IFI16 KO, and control stable cells in a 24-well plate (2 × 10^5^ wells/well) were stimulated with 1 μg/ml dsDNA, 1 μg/ml pcDNA3.1 by transfection for 12 h **(A)**, or HSV-1 at the indicated concentrations for 8 h **(B)**. The stimulated cells were harvested and subjected for RT-qPCR analysis. NS denotes mock stimulation. ^*^p < 0.05, ^**^p < 0.01, ^***^p < 0.001.

To dissect the signaling relationship between porcine cGAS and IFI16 more accurately, we sought to utilize porcine cGAS and IFI16 knockout cells. The CRISPR gRNAs targeting porcine cGAS and IFI16 were designed and cloned into lentiviral vector. The efficacy and specificity of these gRNA expressing lentiviral vectors were verified in transfected cells by Western blotting (Supplementary Figures 2, 3). The packaged lentiviruses were used to infect PAMs and PK15 cells and subjected to puromycin selection to make stable KO cells. As shown in Figure 5, upon stimulation by 45-bp dsDNA and plasmid pcDNA3.1, the downstream IFNβ and ISG56 genes were induced in control PAMs. However, the gene inductions were largely absent in cGAS KO PAMs, which is consistent with the strong cGAS/STING signaling activity observed in transfected cells, suggesting that cGAS is the major DNA sensor in these cells (Figure 5A). Intriguingly, in IFI16 KO PAMs, the DNA-activated IFNβ and ISG56 were both significantly increased compared with those in control PAMs (Figure 5A). HSV-1 stimulated IFNβ and IL8 productions in control PAMs, but the gene inductions were largely decreased in cGAS KO PAMs whereas the same genes were significantly increased in IFI16 KO cells relative to those in control cells (Figure 5B). We also stimulated the PK15 cGAS and IFI16 KO cells with plasmid pcDNA3.1 and HSV-1, and obtained similar results (Supplementary Figures 4A,B). Altogether, the obtained data clearly suggest that porcine IFI16 negatively regulates cGAS signaling and downstream gene transcription.

### Porcine IFI16 Inhibits cGAS Signaling by Competitively Binding With Agonist dsDNA and Adaptor Porcine STING

**Figure 6:**
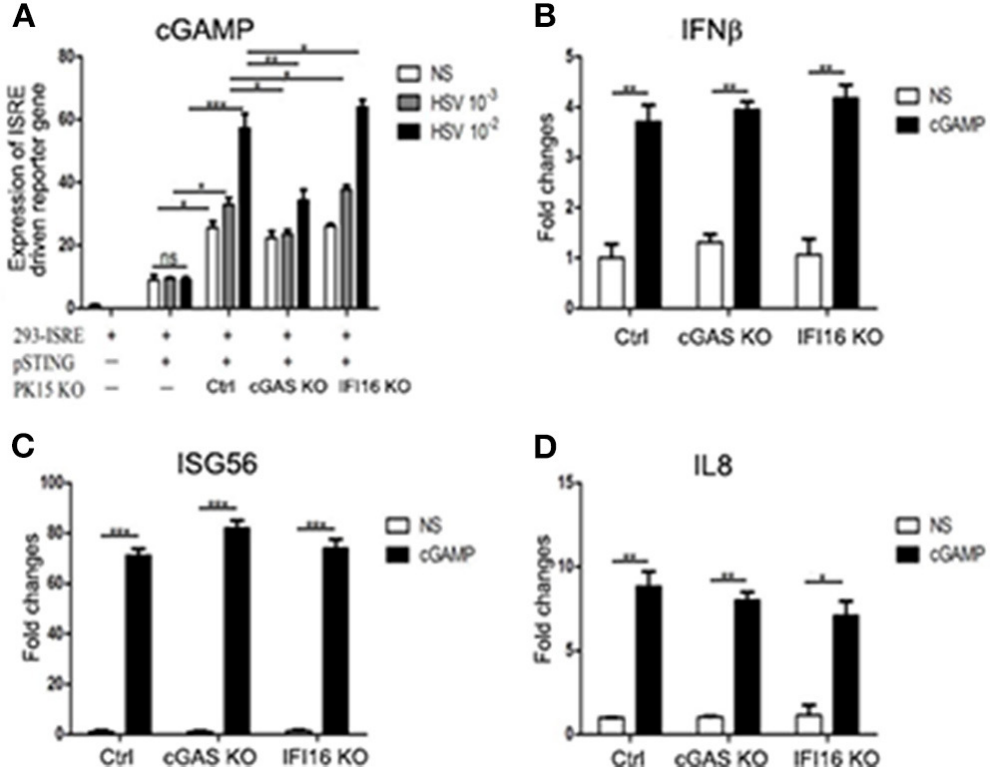
Effects of IFI16 and cGAS KO on HSV-1 stimulated cGAMP production and cGAMP stimulated and STING activated gene transcription. **(A)** ISRE reporter cells in a 24-well plate (2 × 10^5^ cells/well) were transfected with 0.5 μg of pEGFP-pSTING, and 24 h later, the transfected cells were collected by trypsin digestion and cell number was calculated. Simultaneously, PK15 cGAS KO, IFI16 KO, and control stable cells in a 24-well plate (3 × 10^5^ cells/well) were stimulated with HSV-1 at the indicated MOIs for 12 h, then the stimulated cells harvested and cell number calculated. Next, 0.3 × 10^5^ pSTING transfected ISRE reporter cells were incubated with 1.2 × 10^5^ of each type of PK15 stable cells (1:4) in a 24-well plate for 12 h. The incubated pool cells were examined for Fluc and Rluc activities. **(B–D)** The PK15 cGAS KO, IFI16 KO, and control stable cells in a 24-well plate (3 × 10^5^ cells/well) were stimulated with 10 μg/ml 2′3′-cGAMP by transfection for 24 h, and the stimulated PK15 cells were examined for downstream gene transcription by RT-qPCR. NS denotes mock stimulation. ^*^p < 0.05, ^**^p < 0.01, ^***^p < 0.001, ns, not significant.

cGAS is responsible for second messenger 2′5′-cGAMP production, which then directly activates STING for downstream signaling. First, we wondered if porcine IFI16 influences cGAMP production during cGAS activation. We first treated IFI16 KO, cGAS KO, and control stable PK15 cells with HSV-1, and then co-cultured the treated PK15 cells, respectively, with porcine STING transfected ISRE luciferase reporter cells we described before (35). The reporter gene expressions were measured to reflect the cGAMP productions during cGAS activation by HSV-1 infection. As shown in Figure 6A, the control PK15 cells stimulated with HSV-1 produced significant higher levels of reporter gene expression reflecting high level of cGAMP. Compared with control PK15 cells, the cGAS KO PK15 cells had lower amount of cGAMP whereas the IFI16 KO PK15 produced slightly but significantly higher amount of cGAMP compared to control PK15 cells, suggesting that porcine IFI16 might control cGAMP production through cGAS. On the other hand, we would like to know if porcine IFI16 modulated the cGAMP-triggered STING-dependent downstream signaling. The IFI16 KO and cGAS KO PK15 were directly stimulated with cGAMP and downstream gene transcription was examined. The results showed that there was no difference of downstream IFNβ, ISG56, and IL8 levels in IFI16 KO and cGAS KO PK15 cells relative to control cells (Figures 6B–D). It indicates that porcine IFI16 is not likely to regulate cGAMP downstream STING signaling.

**Figure 7:**
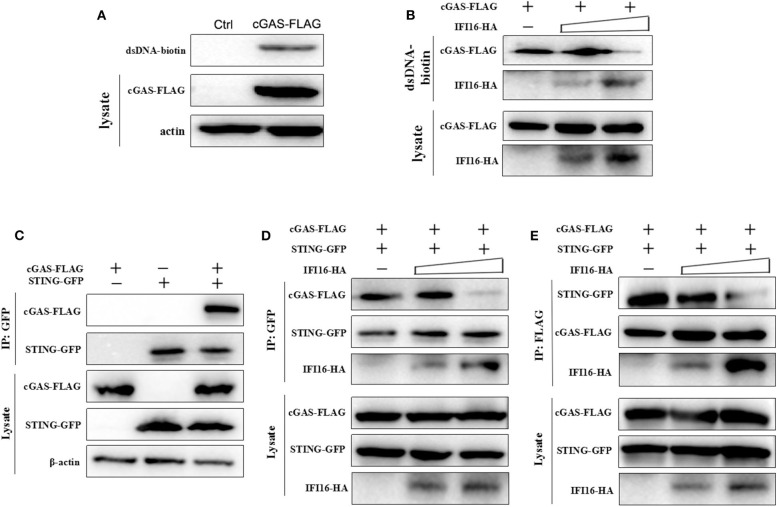
The binding of dsDNA and STING by porcine cGAS in the presence or absence of porcine IFI16. **(A)** 293T cells in a six-well plate (8 × 10^5^ cells/well) were transfected with 0.5 μg of pcGAS or control pcDNA3.1 for 48 h, the transfected cells were lysed, and cell lysates were subjected for dsDNA binding assay with the bound cGAS analyzed by Western blotting using anti-FLAG mAb. **(B)** 293T cells were transfected as in **(A)** without or with 0.5 μg and 1.0 μg porcine IFI16, respectively, and examined for dsDNA binding. **(C)** 293T cells in a six-well plate (8 × 10^5^ cells/well) were transfected with 0.5 μg of pcGAS and 0.5 μg of pSTING for 48 h, and the cell lysates were subjected for Co-IP using anti-GFP antibody and subsequent Western blot analysis. **(D,E)** 293T cells were transfected as in **(C)** without or with 0.5 μg and 1.0 μg porcine IFI16, respectively, and cell lysates were subjected for Co-IP using anti-GFP antibody **(D)** or anti-FLAG antibody **(E)** and subsequent Western blot analysis.

Based on the above results and previous reports (31, 33), we hypothesized that porcine IFI16 might interfere with cGAS for agonist DNA binding to produce cGAMP. Indeed, normally, the biotin-streptavidin conjugated dsDNA agarose could pull down porcine cGAS from cell lysate (Figure 7A), but in the presence of porcine IFI16, the binding of cGAS with dsDNA was impaired (Figure 7B). We also checked the interaction between porcine cGAS and STING in Co-IP and found that there was interaction between these two proteins despite no requirement of this interaction for STING function and signaling (Figure 7C). Further, in the presence of porcine IFI16, the interactions between cGAS and STING became weak as shown by Co-IP in both ways (Figures 7D,E).

**Figure 8:**
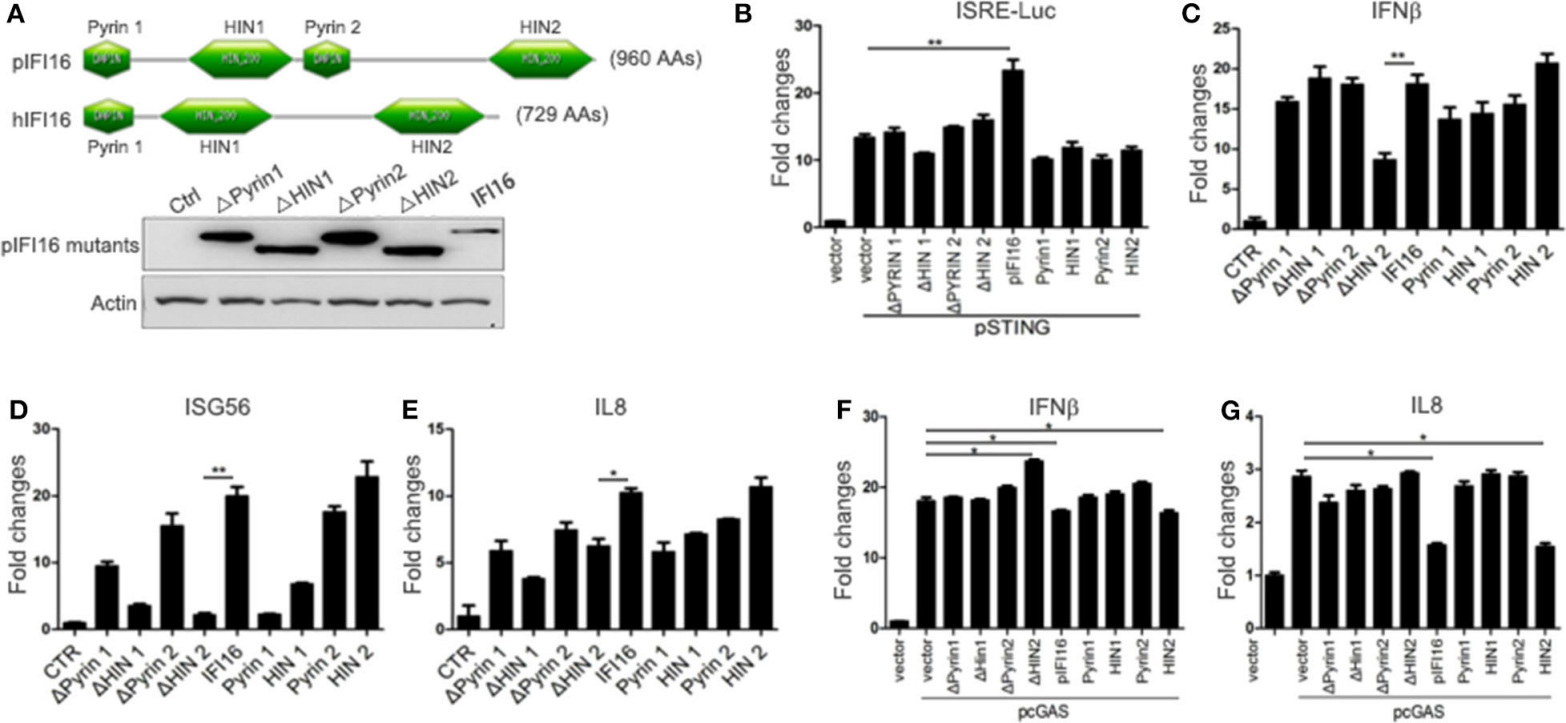
Functional characterization of porcine IFI16 domains. **(A)** The comparison of pIFI16 and hIFI16 domains and expressions of pIFI16 deletion mutants by Western blotting using anti-HA antibody. **(B)** 293T cells in a 96-well plate were transfected with 10 ng of pSTING and 20 ng of each IFI16 mutant together with Fluc and Rluc, and 36 h post-transfection cells were examined for Fluc and Rluc activities. **(C–E)** PK15 in a 24-well plate were transfected with 0.5 μg of each IFI16 mutant for 24 h, and downstream gene transcription was analyzed by RT-qPCR. **(F,G)** PK15 cells in a 24-well plate were transfected with 0.5 μg of pcGAS together with 0.5 μg of each IFI16 mutant for 24 h, and downstream gene transcription was analyzed by RT-qPCR. ^*^p < 0.05, ^**^p < 0.01.

Because of the low sequence identity between porcine and human IFI16, we speculated that species specificity exists. The protein domain prediction by the online software PROSITE from ExPASy (www.ExPASy.org) showed that there is one extra Pyrin domain (Pyrin 2) in porcine IFI16 protein (Figure 8A). To pinpoint the individual roles of each domain in the IFI16 function, we made deletions of each domain and cloned each domain. While the expressions of deletion mutants could be detected by Western blotting, the individual domain expressions were not detectable (Figure 8A). Nevertheless, we proceeded to analyze IFI16 mutants for downstream signaling and gene transcription. In ISRE promoter assay, all the mutants showed activity but much less than full-length IFI16 (Figure 8B). In transfected PK15 cells, all mutants were able to induce downstream IFNβ, ISG56, and IL8 gene transcription, among which domain HIN2 induced close gene transcription to full-length IFI16, while mutant ΔHIN2 had the lowest activity (Figures 8C–E). Correspondingly, similar to full-length IFI16, domain HIN2 significantly inhibited cGAS induced IFNβ and IL8 transcript production, while mutant ΔHIN2 lost the inhibitory ability (Figures 8F,G).

## Discussion

In this study, we functionally assessed the porcine DNA sensors cGAS and IFI16, the two most studied DNA sensors in human and mice, both capable of eliciting STING-dependent signaling (36, 37). IFI16 was identified as a DNA sensor (24); however, IFI16 is a multifunctional protein, once implicated in cell senescence and cell growth control (37, 38). Although IFI16 is a nuclear protein at steady state, as confirmed in our study (Figure 1B), it shuttles between nucleus and cytosol, and its cellular localization appears cell type specific (39). In monocytes and macrophages, it may have significant cytosolic moiety where it exerts canonical DNA sensing function (32, 34), while in non-immune cells such as fibroblasts, it is predominantly localized in nucleus and acts as nuclear DNA sensor, suppressing viral gene expression epigenetically and activating IFN and IFN-stimulated gene (ISG) transcription directly in a non-canonical way (40, 41). More recently, nuclear IFI16 was shown to activate STING via forming complex with p53 and TRAF6 in response to DNA damage (42). The porcine IFI16 has not been studied before despite that the IFI16 from monkey kidney Marc-145 cells was reported to suppress porcine reproductive and respiratory syndrome virus (PRRSV) replication in the cells (43). Regarding porcine cGAS, it has been investigated directly in only one study (44) and for its antiviral properties in several other reports (45–47). We showed here that both porcine cGAS and IFI16 are capable of eliciting IFN signaling dependent on porcine STING, which is exchangeable to human STING (Figures 2, 3). The triple of cytosolic cGAS, IFI16, and STING constitutes the canonical DNA signaling pathway to induce IFN. In the canonical pathway, STING, as an ER resident protein, upon DNA activation traffics from ER to Golgi apparatus and finally to the perinuclear region for degradation. During its trafficking, STING recruits and activates TBK1. In turn, the activated TBK1 phosphorylates IRF3, leading to downstream IFN induction (48).

Importantly, we studied the relationship between porcine IFI16 and cGAS signaling and we did not observe any cooperation between these two DNA receptors. Instead, we found that porcine IFI16 interferes with cGAS for downstream signaling. In comparison, the relation of cGAS and IFI16 in human and mice is quite different. Indeed, several previous studies showed the cooperation between these two DNA receptors during DNA transfection or pathogen infections (30–34). Specifically, human IFI16 was shown to amplify the cGAS-STING canonical pathway to induce IFNβ in response to *Listeria monocytogenes* infection and the subsequent presence of bacterial DNA in the cytosol of human macrophages (32). Mouse IFI16 counterpart p204 cooperated with cGAS to engage in STING-dependent type I IFN production in response to *Francisella novicida* infection and the bacterial DNA in the cytosol of murine macrophages (34). Human IFI16 could positively influence cGAS-STING pathway signaling in macrophages through the increase of second messenger cGAMP production by cGAS and the enhanced recruitment of TBK1 to STING (33), while human IFI16 cooperated with cGAS in keratinocytes by only targeting STING activation (31). Additionally, IFI16 in nucleus could be stabilized by cGAS in human fibroblasts during HSV-1 infection and thus the heightened non-canonical function of IFI16 was obtained (30). In our study, ectopic porcine IFI16 suppressed porcine cGAS/STING-induced phosphorylation of TBK1 and IRF3, ISRE promoter activation, and downstream IFNβ, ISG56, and IL8 transcription (Figure 4). Whereas in porcine IFI16 KO PAMs and PK15 cells, the dsDNA and HSV-1 activated, porcine cGAS-induced downstream genes including IFNβ, ISG56, and IL8 were increased (Figure 5 and Supplementary Figure 4). Therefore, the results clearly showed that porcine IFI16 interferes with cGAS signaling. We speculated the reason that may lead to the discrepancy and thought that could be due to the nature of IFI16, which belongs to the very diverse PYHIN family. In this family, human has four members including IFI16, IFIX, MNDA, and AIM2, whereas mice have 13 members with p204 usually considered as the functional ortholog of human IFI16 (49, 50). In fact, among the 13 mouse members, there are varying degrees of functional redundancy; thus, it is difficult to define the exact functional homolog of IFI16 in mice (49). In contrast, the information in porcine PYHIN family is very limited, and current porcine IFI16 is the only available PYHIN protein that has low identity to human IFI16 and harbors one extra Pyrin 2 domain (Figure 8A). Whether there is another functional homolog of human IFI16 in porcine is now unknown and warrants further investigation.

Regarding the molecular mechanism of action by porcine IFI16 to suppress cGAS signaling, we showed here that both porcine IFI16 and cGAS bind with dsDNA (Figures 7A,B). Furthermore, IFI16 was observed to compete with cGAS for DNA binding (Figures 7A,B). Previous studies showed that both cGAS and IFI16 recognize dsDNA in a sequence-independent way (50, 51); thus, it provides the possibility that these two DNA sensors can compete for dsDNA binding. The competition for agonist dsDNA by IFI16 coincided well with the increased cGAMP level in IFI16 KO cells during HSV-1 infection (Figure 6A). We also found that porcine cGAS and STING interact with each other and that this interaction can be disturbed by porcine IFI16 (Figures 7C–E). However, this disturbance seems not to affect porcine STING activation and downstream gene transcription (Figures 6B–D). Even though STING function was not affected, it could not be excluded that porcine cGAS function might be affected, contributing to reduced cGAMP production. By dissecting the individual domains of porcine IFI16, we found that each domain contributes more or less to IFI16 activity dependent on STING while the HIN2 domain activity is closer to full-length IFI16 (Figures 8B–E). Among the IFI16 domains, the HIN domains are responsible for binding with DNA, while the Pyrin domain mediates homotypic interaction and IFI16 oligomerization (39, 51, 52). In porcine IFI16, HIN2 domain had not only prominent STING dependent activity but also the significant ability to inhibit porcine cGAS activity (Figures 8F,G). Since the HIN2 is the most critical domain for dsDNA binding (48, 53), it also explains well the role of HIN2 in competitive binding agonist dsDNA of cGAS by porcine IFI16 (Figures 7A,B).

**Figure 9:**
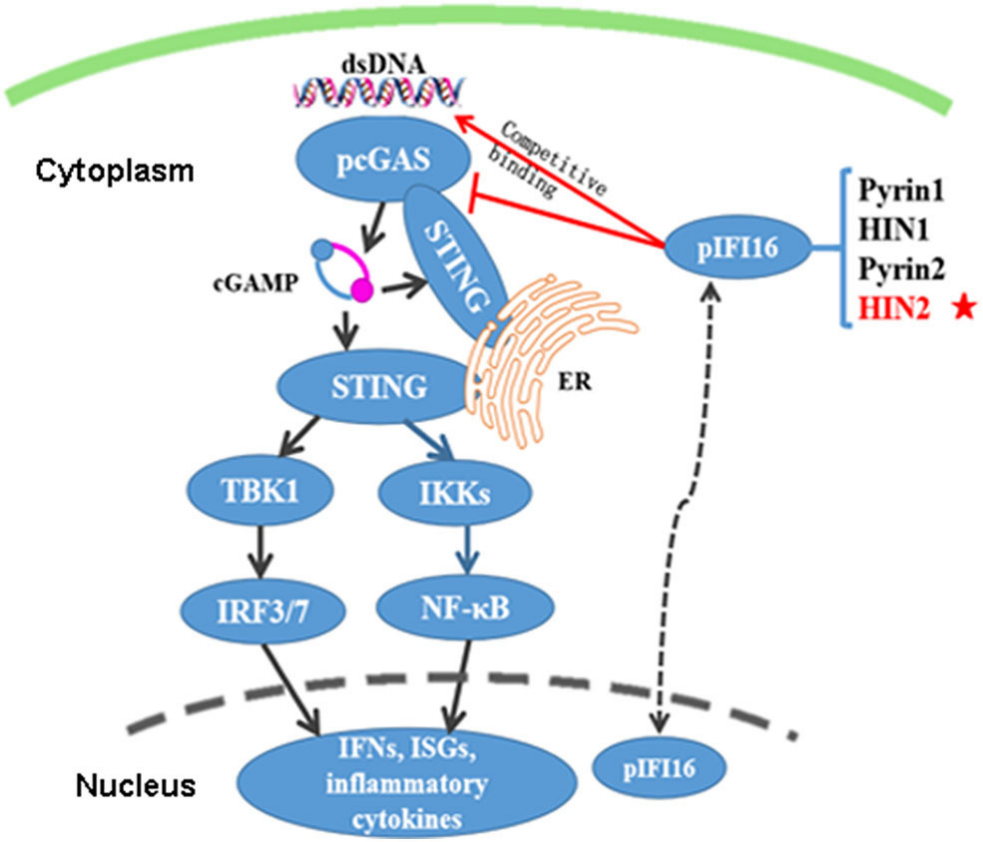
The schematic illustration of porcine cGAS regulation by IFI16. Porcine cGAS, upon recognition of agonist dsDNA, produces second messenger 2′3′-cGAMP, which directly binds with and activates the adaptor STING. The activated STING traffics from ER to Golgi apparatus to the perinuclear region; during the process, it recruits and activates TBK1 that phosphorylates IRF3/7. However, how STING exactly activates NF-κB is still open. In turn, the activated IRF3/7 and NF-κB drive IFN and other gene transcription. Steady-state porcine IFI16 is mainly in the nucleus, but it shuttles into cytosol where it competes with cGAS for binding to agonist DNA and STING mainly via its HIN2 domain, thus modulating the cGAMP production and downstream signaling.

Collectively, this study isolated two porcine DNA sensors cGAS and IFI16, confirmed their STING-dependent IFN-inducing activity, analyzed the relation between these two DNA sensors, and explored the mechanism of action utilized by porcine IFI16 to regulate cGAS signaling (Figure 9), and therefore revealed unique insights into innate immune biology in the pig, which is the promising model for human diseases.

## Data Availability Statement

All datasets generated for this study are included in the article/Supplementary Material.

## Author Contributions

JZ conceived and designed the experiments. WZ, RZ, SL, SH, JL, and MZ performed the experiments. WZ, NC, HC, FM, and JZ analyzed the data. WZ and JZ wrote the paper. All authors contributed to the article and approved the submitted version.

## Conflict of Interest

The authors declare that the research was conducted in the absence of any commercial or financial relationships that could be construed as a potential conflict of interest.

## Supplementary Material

Supplementary Figure 1 The pcDNA-cGAS, pcDNA-IFI16, pEGFP-STING (0.5 μg each) were transfected into 293T cells (4 × 10^5^ cells/well on coverslips in 12-well plate) using lipofectamine 2000. Forty-eight hours post transfection, the protein expressions were examined by fluorescence microscopy after fixation and staining.

Supplementary Figure 2 The validation of efficacy and specificity of gRNA lentiviral plasmids targeting porcine cGAS. 293 T cells (5 × 10^5^ cells/well) were transfected with 0.5 μg porcine cGAS or IFI16 together with 0.5 and 1 μg cGAS gRNAs, respectively, 48 h later, the transfected cells were analyzed by Western blotting using anti-HA antibody.

Supplementary Figure 3 The validation of efficacy and specificity of gRNA lentiviral plasmids targeting porcine IFI16. 293 T cells (5 × 10^5^ cells/well) were transfected with 0.5 μg porcine IFI16 or cGAS together with 0.5 and 1 μg IFI16 gRNAs, respectively, 48 h later, the transfected cells were analyzed by Western blotting using anti-HA antibody.

Supplementary Figure 4 Effects of IFI16 and cGAS KO on DNA and HSV-1 stimulated gene transcription. PK15 cGAS KO, IFI16 KO, and control stable cells in 24-well plate (2 × 10^5^ wells/well) were stimulated with 1 μg/ml pcDNA3.1 by transfection for 12 h **(A)**, or HSV-1 at the indicated concentrations for 8 h **(B)**. The stimulated cells were harvested and subjected for RT-qPCR analysis. NS denotes mock stimulation. ^*^*p* < 0.05, ^**^*p* < 0.01, ^***^*p* < 0.001.

Supplementary Table 1 The PCR primers used for gene cloning and mutations.

Supplementary Table 2 The CRISPR gRNA encoding DNA sequences for porcine cGAS and IFI16.

Supplementary Table 3 Primers for RT-PCR and RT-qPCR in this study.
