# Supplementary figures and images for: Porcine IFI16 Negatively Regulates cGAS Signaling Through the Restriction of DNA Binding and Stimulation
Source: Front Immunol. 2020 Aug 14;11:1669. doi: 10.3389/fimmu.2020.01669 (PMC7456882; doi:10.3389/fimmu.2020.01669)

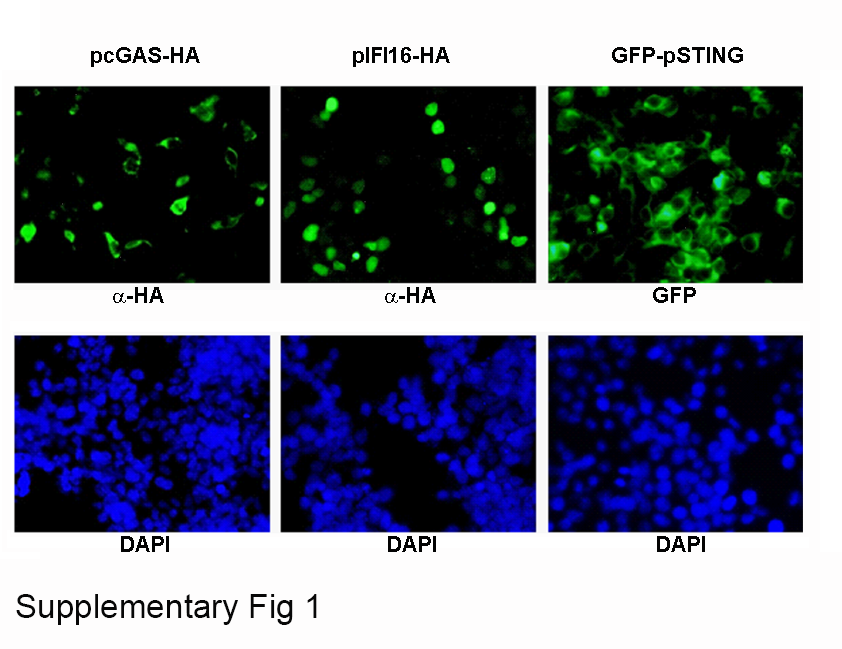

Supplement: Supplementary Figure 1 — The pcDNA-cGAS, pcDNA-IFI16, pEGFP-STING (0.5 μg each) were transfected into 293T cells (4 × 10^5^ cells/well on coverslips in 12-well plate) using lipofectamine 2000. Forty-eight hours post transfection, the protein expressions were examined by fluorescence microscopy after fixation and staining. [file Image_1.TIF]

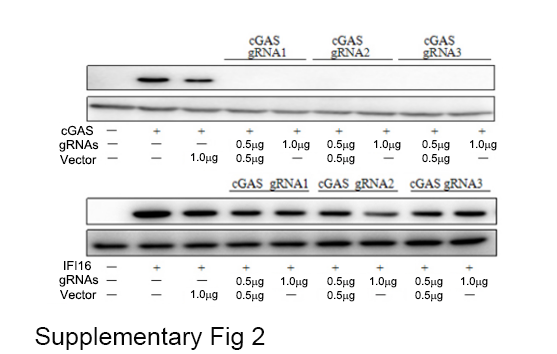

Supplement: Supplementary Figure 2 — The validation of efficacy and specificity of gRNA lentiviral plasmids targeting porcine cGAS. 293 T cells (5 × 10^5^ cells/well) were transfected with 0.5 μg porcine cGAS or IFI16 together with 0.5 and 1 μg cGAS gRNAs, respectively, 48 h later, the transfected cells were analyzed by Western blotting using anti-HA antibody. [file Image_2.TIF]

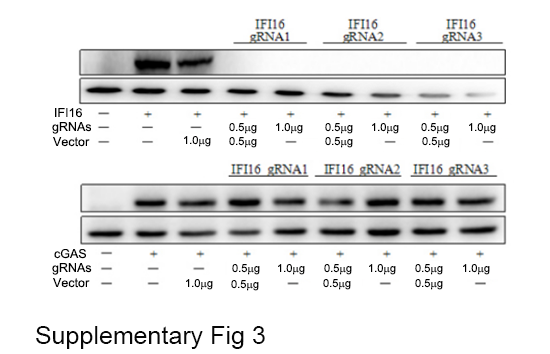

Supplement: Supplementary Figure 3 — The validation of efficacy and specificity of gRNA lentiviral plasmids targeting porcine IFI16. 293 T cells (5 × 10^5^ cells/well) were transfected with 0.5 μg porcine IFI16 or cGAS together with 0.5 and 1 μg IFI16 gRNAs, respectively, 48 h later, the transfected cells were analyzed by Western blotting using anti-HA antibody. [file Image_3.TIF]

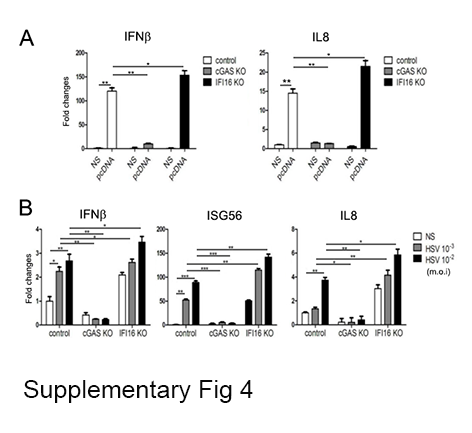

Supplement: Supplementary Figure 4 — Effects of IFI16 and cGAS KO on DNA and HSV-1 stimulated gene transcription. PK15 cGAS KO, IFI16 KO, and control stable cells in 24-well plate (2 × 10^5^ wells/well) were stimulated with 1 μg/ml pcDNA3.1 by transfection for 12 h (A), or HSV-1 at the indicated concentrations for 8 h (B). The stimulated cells were harvested and subjected for RT-qPCR analysis. NS denotes mock stimulation. *p < 0.05, **p < 0.01, ***p < 0.001. [file Image_4.TIF]
