# Supplementary material for: Porcine IFI16 Negatively Regulates cGAS Signaling Through the Restriction of DNA Binding and Stimulation
Source: Front Immunol. 2020 Aug 14;11:1669. doi: 10.3389/fimmu.2020.01669 (PMC7456882; doi:10.3389/fimmu.2020.01669)
Supplement: Supplementary Table 1 — The PCR primers used for gene cloning and mutations. [file Data_Sheet_1.docx]

**Supplementary Table 1**, The PCR primers used for gene cloning and mutations

| Gene names | Primer names | Primer sequences (5’→3’) | Amplication sizes (bp) |
| --- | --- | --- | --- |
| pcGAS | cGAS-F | CCAGCC**ATG**GCGGCCCGGC | 1502  (cloning) |
|  | cGAS-R | TTCGCGATATCCCAAAAAACTGGAAATCCATTGTTTCTTTCATATTCAATTTGCTTTGACAGAAATTCTTTACTTGG |  |
|  | cGAS-f | TGGAGGCTTTCCTTCTCTCA | 230  (detection) |
|  | cGAS-r | TGAGGGTCCTGGGTACAGAC |  |
| pIFI16 | IFI16-F | TTCGCGTCGAC**ATG**ACGAATAAATACAAGAAAATCATTCTGC | 2902  (cloning) |
|  | IFI16-R | TTCGCGATATCCCTGGCCTTGATGACCTTGATG |  |
|  | IFI16-f | GTTCCCAGGAATTACTTGTGTC | 316  (detection) |
|  | IFI16-r | GGAGACAGACTGAGTCTGC |  |
| ∆Pyrin-1  (∆ AAs 1-89) | Forward ∆P1 | TTCGCGCTAGC**ATG**GCAAGGGAAATTGGAGCAGAA | 2638 |
|  | Reverse | TTCGCGATATCCCTGGCCTTGATGACCTTGA |  |
| ∆HIN200-1  (∆ AAs 196-395) | IFI16-F | TTCGCGCTAGC**ATG**ACGAATAAATACAAGAAAATCATTCTGC | 596 |
|  | Reverse ∆H1 | CTGGCTTTTCTGGTTCTTGGG |  |
|  | Bridge ∆H1 | CCTACCCAAGAACCAGAAAAGCCAGAAGACTAAGAAAAGAAGGACCAATTTAATTCAGAAAGT |  |
|  | Forward ∆H1 | AAGACTAAGAAAAGAAGGACCAATTTAATTCA | 1706 |
|  | Reverse | TTCGCGATATCCCTGGCCTTGATGACCTTGA |  |
| ∆Pyrin-2  (∆ AAs 414-501) | IFI16-F | TTCGCGCTAGC**ATG**ACGAATAAATACAAGAAAATCATTCTGC | 1250 |
|  | Reverse ∆P2 | TGCAGCTGATTCTCCAACTTTC |  |
|  | Bridge ∆P2 | AATTCAGAAAGTTGGAGAATCAGCTGCAGTTTTAAGGAGACTCAATGCAAAAGAAACCG |  |
|  | Forward ∆P2 | GTTTTAAGGAGACTCAATGCAAAAGAAA | 1388 |
|  | Reverse | TTCGCGATATCCCTGGCCTTGATGACCTTGA |  |
| ∆HIN200-2  (∆ AAs 758-960) | IFI16-F | TTCGCGCTAGC**ATG**ACGAATAAATACAAGAAAATCATTCTGC | 2293 |
|  | Reverse ∆H2 | TTCGCGATATCCTTCAATCTTGGTTTCTCCTGTCTC |  |
| Pyrin1  (AAs 1-88) | F-P1 | GGGAGACCCAAGCTGGCTAGCGCCACC**ATG**ACGAATAAATACAAGAAAATCATTCTGC | 312 |
|  | R-P1 | GTATGGGTAGCTGGTGATATCTTTTAGCTTTTCCTTTCTAAATTCTTTTACAGT |  |
| HIN200-1  (AAs 196-395) | F-H1 | GGGAGACCCAAGCTGGCTAGCGCCACC**ATG**GTCCAACATAAGGGAGCTGC | 628 |
|  | R-H1 | GTATGGGTAGCTGGTGATATCGATGACCTGGATGAAGCTGTG |  |
| Pyrin2  (AAs 414-501) | F-P2 | GGGAGACCCAAGCTGGCTAGCGCCACC**ATG**GGGAATGAATTCAAGAGAATTGT | 312 |
|  | R-P2 | GTATGGGTAGCTGGTGATATCTTTTAACTTCTCCTTTTGAAGAGGTTTAACA |  |
| HIN200-2  (AAs 758-957) | F-H2 | GGGAGACCCAAGCTGGCTAGCGCCACC**ATG**GTGGTGCCGAAAGAAGCTT | 628 |
|  | R-H2 | GTATGGGTAGCTGGTGATATCGATGACCTTGATGAAACTGTGAATTACA |  |

**Note:** The positions of gene fragments are indicated, the start codons are shown in bold, and the restriction endonuclease sites are underlined.

**Supplementary Table 2**, The CRISPR gRNA encoding DNA sequences for porcine cGAS and IFI16

| Target genes | gRNA names | gRNA sequence(5’→3’) |
| --- | --- | --- |
| pcGAS | c-gRNA1-F | CACCGCCGCTTCTGAGATTTCGTGG |
|  | c-gRNA1-R | AAACCCACGAAATCTCAGAAGCGGC |
|  | c-gRNA2-F | CACCGAGAAGCCGCAGGTACGCACG |
|  | c-gRNA2-R | AAACCGTGCGTACCTGCGGCTTCTC |
|  | c-gRNA3-F | CACCGGAGGCTCTACCCTTTCGGA |
|  | c-gRNA3-R | AAACTCCGAAAGGGTAGAGCCTCC |
| IFI16 | I-gRNA1-F | CACCGAGATGGCAGATTAATATGTG |
|  | I-gRNA1-R | AAACCACATATTAATCTGCCATCTC |
|  | I-gRNA2-F | CACCGGCAGATTAATATGTGTGGT |
|  | I-gRNA2-R | AAACACCACACATATTAATCTGCC |
|  | I-gRNA3-F | CACCGAAACATGTTCCGATATGCA |
|  | I-gRNA3-R | AAACTGCATATCGGAACATGTTTC |

**Supplementary Table 3**, Primers for RT-PCR and qRT-PCR in this study

| Gene Names | primer sequence (5’-3’) | Sizes (bp) |
| --- | --- | --- |
| hIFNβ | TGGGAGGATTCTGCATTACC | 191 bp |
|  | CAGCATCTGCTGGTTGAAGA |  |
| hISG56 | CGCTATAGAATGGAGTGTCCA | 68 bp |
|  | TTTCCTCCACACTTCAGCA |  |
| hIL8 | GTTTTTGAAGAGGGCTGAGAATTC | 108 bp |
|  | CATGAAGTGTTGAAGTAGATTTGCTTG |  |
| hRPL32* | CAACATTGGTTATGGAAGCAACA | 79 bp |
|  | TGACGTTGTGGACCAGGAACT |  |
| pIFNβ | TGAGCATTCTGCAGTACCTGA | 116 bp |
|  | CCGGAGGTAATCTGTAAGTCTGT |  |
| pISG56 | ATGGGAGTTGGTCATTCAAGA | 127 bp |
|  | CAGGTGTTTCACATAGGCCA |  |
| pISG60 | CCCGACAACCCAGAATTCTCCT | 597 bp |
|  | AGAGCGCTGATGAAGTTGTTGC |  |
| pIL8 | CTGCAGTTCTGGCAAGAGTAAGT | 108 bp |
|  | CACTCTCAATCACTCTCAGTTCCT |  |
| pβ-actin* | ATGAAGATCAAGATCATCGCG | 116 bp |
|  | TCGTACTCCTGCTTGCTGATC |  |

* Housekeeping genes
